# The Effect of Mycotoxins and Their Mixtures on Bovine Spermatozoa Characteristics

**DOI:** 10.3390/toxins15090556

**Published:** 2023-09-06

**Authors:** Dorit Kalo, Paz Mendelson, Alisa Komsky-Elbaz, Hillary Voet, Zvi Roth

**Affiliations:** 1 Department of Animal Sciences, Robert H. Smith Faculty of Agriculture, Food and Environment, The Hebrew University, Rehovot 7610001, Israel; 2 Department of Agricultural Economics and Management, Robert H. Smith Faculty of Agriculture, Food and Environment, The Hebrew University, Rehovot 7610001, Israel

**Keywords:** mycotoxins, spermatozoa, mycotoxin mixtures, mycotoxin interactions, bovine

## Abstract

There is growing concern about the effects of mycotoxins on mammalian reproduction. Although the effects of single mycotoxins have been well documented, the impact of their mixtures on spermatozoon quality is less known. Here, frozen-thawed semen (*n* = 6 bulls) was in-vitro-cultured (2 h) without (control) or with (i) a single mycotoxin [zearalenone (ZEN), ochratoxin A (OTA), toxin 2 (T2), and diacetoxyscirpenol (DAS)] in a dose-response manner; (ii) binary mixtures (OTA + T2, OTA + ZEN, OTA + DAS, ZEN + T2, DAS + T2 and ZEN + DAS); or (iii) ternary mixtures (OTA + DAS + T2, OTA + ZEN + T2, and ZEN + DAS + T2). Then, the spermatozoa quality was characterized according to its plasma- and acrosome-membrane integrity, mitochondrial membrane potential, and oxidation status by a flow cytometer. Exposure to single mycotoxins or binary mixtures did not affect the spermatozoa characteristics. However, exposure to the ternary mixtures, OTA + DAS + T2 and OTA + ZEN + T2, reduced (*p* < 0.05) the mitochondrial membrane potential relative to the control. In addition, OTA + ZEN + T2 increased (*p* < 0.05) the proportion of spermatozoa with reactive oxygen species relative to the control. The most suggested interaction effect between the mycotoxins was found to be an additive one. A synergistic interaction, mainly regarding the oxidation status of the spermatozoa, was also found between the mycotoxins. The current study sheds light on the potential risk of exposing spermatozoa to a mycotoxin mixture.

## 1. Introduction

Mycotoxins are secondary metabolites produced by the genera Aspergillus, Fusarium, and Penicillium [1], which can colonize cereal grains and animal feed. Mycotoxins are considered a “natural product” that can evoke, at a low concentration, a toxic response [2]. Mycotoxin production is affected by temperature, humidity, the presence of fungicides and/or fertilizers, geographical location, genetic requirements, nutritional factors, and insect infestation [3,4]. Mycotoxins can be produced at any stage of growth, processing, transport, and storage [4,5]. For instance, mycotoxins can enter the food chain through contaminated cereal grains, which are sources of energy and protein for all classes of farm livestock. Moreover, it is estimated that about 25–50% of commodities are contaminated with mycotoxins [4,6]. About 400 mycotoxins have been identified so far, which differ in their structure and biochemical effects, thereby leading to a diverse range of toxic effects [7,8]. In that respect, legislative guidance for several mycotoxin ranges in animal feed was set up by the FAO Food and Nutrition, European Food Safety Authority (EFSA), and the EU Commission recommendation [9,10,11].

For both human and animal health, various mycotoxins are a major concern [3,7,12,13]. In particular, trichothecenes, ochratoxins, aflatoxins, zearalenone, fumonisins, patulin, and citrinin are the most toxic and are considered a major concern to agriculture, human, and animal health [3,7]. For the current study, four known mycotoxins, namely, zearalenone (ZEN), ochratoxin A (OTA), toxin 2 (T2), and iacetoxyscirpenol (DAS), all of which were detected in the feedstuff of dairy cattle, were chosen [5,14]. ZEN is a non-steroidal estrogenic that resembles 17β-estradiol activity; thus, it is known as an estrogenic mycotoxin [4,15]. Studies in male rodents reported that ZEN impairs hormonal balance and causes reproduction and fertility disorders [16]. Once ZEN enters the body, it undergoes bio-transformations that result in two main metabolites: α-zearalenol and β-zearalenol. OTA is considered to be the most toxic mycotoxin within the ochratoxin family members [1,7]. Previous studies in humans, fishes, quails, dogs, pigs, mice, rats, and monkeys characterized OTA as a nephrotoxin, an immune suppressant, a potent teratogen, and a carcinogen [17,18,19]. OTA was found to be involved in several cellular mechanisms, including inhibition of protein synthesis, induction of membrane lipid peroxidation, and inhibition of mitochondrial respiration [18,20]. T2 and DAS mycotoxins belong to the trichothecenes A type family; of all the family members (150), they are considered the most toxic mycotoxins from [7,21]. Both T2 and DAS have been found to affect human and animal health [10,22,23], characterized by deleterious effects on DNA and RNA synthesis, cell division, membrane structure, and mitochondrial function [22,24,25]. Although ZEN and T2 have been reported to be associated with infertility in cattle [26], the mycotoxins OTA and DAS have been considered testicular toxins only in humans and mice [10,27]. In cattle, OTA is not considered to be a reproductive toxicant when fed alone and at relatively low levels [26]. Nevertheless, the latter statement is mainly relevant to female cows rather than males and emphasizes the risk of exposure to offspring. There is a lack of knowledge regarding the effect of these mycotoxins on the male, in particular, on the bull spermatozoa. 

Considering that more than one fungus can produce more than one mycotoxin and that several fungi can be found at the same time in the feed, there is a high risk of the co-existence of several mycotoxins. Most of the mycotoxin studies have examined the effect of a single mycotoxin; however, the toxic effect of combined mycotoxins has been less studied. Although interpreting a combined toxic effect is highly challenging, three types of interactions have been defined to date. These interactions include synergistic, additive, or antagonistic, which correspond to a greater, equal, or lower effect than the summed effects of individual mycotoxins, respectively [28,29]. It is commonly assumed that mycotoxins share a similar mechanism of action and that they will interact in a synergistic or additive manner [30]. However, this is not always the case since other factors, such as the mycotoxin concentration, the type of targeted cells, or the animal model, can influence the combined toxic effect. It is worth mentioning that the applied statistical method plays a pivotal role in interpreting the mycotoxin interactions [31].

The reduction in male fertility, for both humans and animals, is considered to be a worldwide problem and is associated with semen and spermatozoa quality [32]. Among the potential causes for reduced semen quality are genetic diseases, polygenetic risk factors, geographical aspects, as well as reproductive, pathological, and environmental stressors [33,34,35]. With respect to the latter, environmental toxins, mainly mycotoxins, are potential hazards that are involved in male fertility decline [13,36,37,38]. In our recent study, we reported that in vitro exposure of bovine spermatozoa to the mycotoxin, aflatoxin B1, at 10 µM during a 4 h capacitation impaired the spermatozoa’s viability, mitochondrial membrane potential, as well as the acrosome and DNA integrity [39]. These alterations were associated with lower fertilization competence, manifested by a lower proportion of oocytes that were fertilized and cleaved to two- to four-cell-stage embryos [39]. Moreover, aflatoxin B1 induced alterations in the proteomic profile of the treated spermatozoa [40]. For the current study, we hypothesized that direct exposure to single mycotoxins or their mixtures would affect the spermatozoa quality. Using the bovine model and conducting a dose-response assay, we examined the effects of single mycotoxins (DAS, ZEN, T2, and OTA) on the spermatozoa’s cellular features, including plasma- and acrosome-membrane integrity, mitochondrial membrane potential, and oxidation status. In addition, the effect of a mixture of two and three mycotoxins has been examined. Accordingly, a potential interaction between two or more mycotoxins was also suggested. The findings reported herein provide new information and enhance our understanding of the impact of mycotoxins on spermatozoa characteristics.

## 2. Results

### 2.1. Solvent Toxicity Examination

First, a control examination was performed to examine the effect of the solvent (i.e., methanol) on the spermatozoa characteristics. Spermatozoa were in-vitro-cultured for 2 h at 38.5 °C without (untreated) or with methanol at 4% (*v*/*v*) (i.e., control), which is equivalent to the highest concentration of the dissolved mycotoxin used in the study. At the end of the 2 h exposure, samples were immediately analyzed by a flow cytometer to assess the spermatozoa’s cellular features, detailed in the Materials and Methods (Section 5.2).

The findings indicated that methanol at the examined concentrations did not affect the examined parameters relative to the untreated spermatozoa (Table 1). In light of these findings, the methanol group served as a control for the subsequent experiments.

### 2.2. Dose-Response Study—The Cytotoxicity of Single Mycotoxins

The first set of experiments included a dose-response study in order to examine the cytotoxicity of a single mycotoxin. Spermatozoa (from 6 bulls, where each bull represents a biological replicate) were incubated without (control) or with a single mycotoxin (DAS, ZEN, OTA, and T2) in a warm bath at 38.5 °C for 2 h. Then, the spermatozoa were immediately analyzed by a flow cytometer to assess the spermatozoa cellular features, detailed in the Materials and Methods (Section 5.2). Overall, exposing spermatozoa to each mycotoxin did not affect the proportion of viable spermatozoa at any of the chosen concentrations relative to the control. In addition, no single mycotoxin affected the mitochondrial membrane potential, the oxidation status of viable spermatozoa, or the acrosomal membrane integrity (i.e., the proportion of spermatozoa with an intact or damaged acrosomal membrane) compared with the control. The findings are summarized in Table 2.

### 2.3. Effect of Mixture-II—Cytotoxicity of the Binary Mixtures of Mycotoxins

A second set of experiments was conducted to examine the effect of the binary combination of two mycotoxins. Spermatozoa were obtained from three bulls, a subset of bulls from the set of the six bulls used for the single mycotoxin study (2.2), where each bull represents a replicate. Spermatozoa were incubated without (control) or with a binary study (2.2) mycotoxin combination (ZEN + OTA, ZEN + T2, ZEN + DAS, OTA + DAS, OTA + T2, and DAS + T2) in a warm bath at 38.5 °C for 2 h. Then, the spermatozoa were immediately analyzed by the flow cytometer to assess the spermatozoa cellular features, detailed in the Materials and Methods (Section 5.2). For each binary combination, three concentrations (the lowest, intermediate, and highest) were chosen for each mycotoxin (ZEN: 0.1, 1, 10 ppm; OTA: 0.05, 0.25, 10 ppm; T2: 0.01, 0.1, 0.5 ppm, and DAS: 0.01, 0.1, 0.5 ppm). Overall, exposing spermatozoa to binary combinations of mycotoxins did not affect the proportion of viable spermatozoa, the mitochondrial membrane potential, the oxidation status of viable spermatozoa, and the acrosome-membrane integrity at all concentrations relative to the control group (Table 3).

#### Estimated Interactions between Two Mycotoxins

Considering the comparison between the expected effect relative to the measured one for the binary mixtures, the most prominent interaction effect was found to be an additive effect (Table 4a–f). Nevertheless, synergistic and antagonistic interactions are also suggested, as described below. With respect to the proportion of viable spermatozoa, a synergistic effect is suggested between OTA + DAS (*p* = 0.02) and OTA + T2 (*p* = 0.05) at the intermediate concentration (Table 4b,c, respectively). This was reflected by the greater effect of the measured effect relative to the calculated expected effect of these binary combinations. With respect to the effect on the mitochondrial membrane potential, exposing spermatozoa to binary mixtures resulted in an additive effect in all the examined concentrations (Table 4a–f). With respect to acrosome membrane integrity, a synergistic effect is suggested between OTA and DAS at high concentrations (*p* = 0.05) regarding the proportion of spermatozoa with an intact acrosome (Table 4b). In parallel, an antagonistic effect was found between these mycotoxins regarding the proportion of spermatozoa with damaged acrosome (high concentrations; *p* = 0.007). Regarding the spermatozoa’s oxidation status, a synergistic effect is suggested between ZEN and T2 (high concentrations; *p* = 0.03). This was reflected by a smaller expected effect relative to the measured effect on the proportion of viable spermatozoa with reactive oxygen species (ROS+) in these binary mixtures (Table 4e). 

### 2.4. Effect of Mixture-III—The Cytotoxicity of Ternary Mixtures of Mycotoxins

A third set of experiments was conducted to examine the effect of a ternary combination on the spermatozoa’s cellular features. Spermatozoa were obtained from three bulls, a subset of bulls from the set of the six bulls used for the single mycotoxin study (2.2), where each bull represents a replicate. Spermatozoa were incubated without (control) or with a ternary mycotoxin combination (ZEN + OTA + T2, ZEN + T2 + DAS and T2 + DAS + OTA) in a warm bath at 38.5 °C for 2 h. Then, the spermatozoa were immediately analyzed by a flow cytometer to assess the spermatozoa’s cellular features, detailed in the Materials and Methods (Section 5.2). For each ternary combination, the intermediate concentration for each mycotoxin was used (ZEN: 1 ppm; OTA: 0.25 ppm; T2: 0.1 ppm, and DAS: 0.1 ppm). 

**Figure 1:**
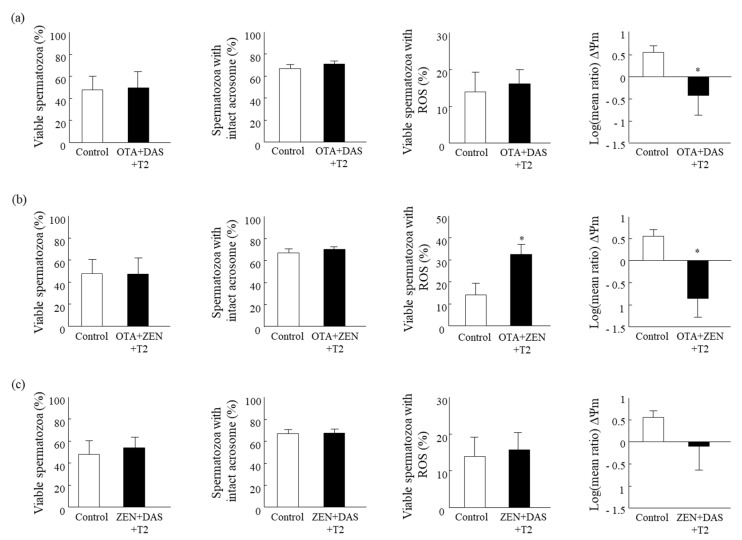
Effect of three mycotoxin mixtures on the spermatozoa features. Presented is the proportion of viable spermatozoa, spermatozoa with an intact acrosome membrane, viable spermatozoa with ROS+, and the mitochondrial membrane potential (i.e., the log(mean ratio of the polarized/depolarized), followed by exposure to an OTA + DAS + T2 mixture (**a**), an OTA + ZEN + T2 mixture (**b**), or a ZEN + DAS + T2 mixture (**c**). For each mixture, the data include 3 replicates with 5000 spermatozoa for each. Concentrations of mycotoxins within the mixtures were as follows: 1 ppm ZEN, 0.25 ppm OTA, 0.1 ppm T2, and 0.1 ppm DAS. Presented are the values of the means ± S.E.M. * An asterisk above a bar indicates a significant difference, *p* < 0.05, relative to the control.

Exposing spermatozoa to an OTA + DAS + T2 mixture reduced the mitochondrial membrane potential relative to the control group (*p* < 0.05; Figure 1a). However, this mixture did not affect the proportion of viable spermatozoa, the proportion of spermatozoa with an intact acrosome membrane, or the proportion of ROS+ spermatozoa (Figure 1a). Exposing spermatozoa to a ZEN + OTA + T2 mixture decreased the mitochondrial membrane’s potential and increased the proportion of ROS+ spermatozoa relative to the control (*p* < 0.05; Figure 1b). No effect was recorded regarding the proportion of viable spermatozoa or the proportion of spermatozoa with an intact acrosome membrane relative to the control (Figure 1b). A mixture of ZEN + DAS + T2 did not affect any of the examined cellular features (Figure 1c). 

#### Estimated Interactions between Three Mycotoxins

The prominent suggested interaction between three mycotoxins was found to be additive. This was true for the effect of all ternary mixture combinations on the mitochondrial membrane potential and the acrosome membrane integrity (Table 5). In addition, a synergistic effect is suggested between DAS, T2, and OTA regarding the proportion of viable spermatozoa (*p* = 0.02; Table 5), with a greater effect of the measured vs. the expected effects. 

## 3. Discussion

The negative impact of mycotoxins on reproductive health has been previously documented for humans and animals. Although most of the studies examined a single type of mycotoxin, the current study provides new evidence of the harmful effect of mycotoxin mixtures on bovine spermatozoa. There is a lack of data regarding the actual mycotoxin levels in the bull’s circulation, in particular, within the testicular tissue, i.e., the natural environment of the spermatozoa. Limited information indicates some residues or traces of mycotoxins, mainly ZEN and OTA, in serum, urine, milk, and tissues of the cow [41,42,43]. Accordingly, in the current study, we examined the effect of mycotoxins on the bull spermatozoa at a range of admitted limits to be in the feed. Exposing spermatozoa to a single mycotoxin (ZEN, DAS, T2, or OTA), as well as to binary mycotoxin mixtures, did not affect any of the examined cellular features. On the other hand, exposing spermatozoa to ternary mixtures of mycotoxins affected mitochondrial functioning, manifested by a lower membrane potential and a higher ROS expression. The major interactions were additive, and some were synergistic, suggesting that not only the concentration but also the number of mycotoxins and the interaction between them play a role in the resulting toxic effect. 

### 3.1. Evaluation of the Spermatozoa Quality 

Within the common andrology methods used to evaluate semen and/or spermatozoa quality, spermatozoa membrane integrity was found to be a more relevant marker for fertilizing capacity [44,45]. Membrane integrity is vital for spermatozoa functioning, such as interaction with the oocyte and the female reproductive tract [46,47]. Evaluation of the membrane viability is generally determined by examining the permeability and the integrity of the membrane [44,48,49]. In our study, we found that exposure of the spermatozoa to single mycotoxins or in binary or ternary mixtures did not affect the membrane integrity. In agreement, a long-term (1 week) in vitro exposure of boar spermatozoa to ZEN at 10 and 1000 µg/L did not affect their membrane integrity [50]. Furthermore, in vitro exposure of boar spermatozoa to OTA at 10 or 100 µM for 24 h had no effect on the percentage of viable spermatozoa [51]. In contrast, in vitro exposure of bovine spermatozoa to ZEN or T2 (0.5 mM to 0.01 mM) for 1 h induced severe damage to the membrane [52]. Recently, it was reported that in vitro exposure of buffalo spermatozoa to ZEN at 2000 ng/mL for 2 h impaired their viability, whereas lower concentrations (i.e., 10, 100, and 1000 nb/mL did not [53]. Similarly, in vitro exposure of boar semen to 62.8 µM ZEN for 1, 2, 3, and 4 h reduced the spermatozoa viability [54]. Moreover, in vitro exposure of boar semen to ZEN at 1 × 10^−8^ to 1 μM for 24–48 h reduced the spermatozoa viability and negatively affected the chromatin’s structural stability [55]. A reduction in spermatozoa viability is associated with a lower probability of fertilizing the oocyte since a higher percentage of dead spermatozoa will lower the probability. Here, we reported that mycotoxins did not affect the proportion of viable sperm; thus, it can generally be speculated that direct exposure of spermatozoa to mycotoxins at the time of artificial insemination may not impair the spermatozoa’s ability to fertilize the oocyte. Nevertheless, other effects on cellular features that are associated with fertility competence, such as motility, DNA damage, acrosome membrane integrity, and mitochondria functioning may be involved. 

The membrane integrity of the acrosome also plays a role in fertilization success since an intact acrosome is required when the spermatozoa reach the site of fertilization [56]. Here, we report that exposing spermatozoa to a single or a mixture of mycotoxins did not impair the integrity of the acrosome membrane. In agreement, in our previous study, we found that in vitro exposure of fresh bovine spermatozoa to aflatoxin B1 at 0.1, 1, 10, or 100 µM did not affect the acrosome integrity [39]. Similarly, a previous study reported that in vitro exposure of boar spermatozoa to 10 or 100 µM OTA for 24 h did not affect the acrosome membrane integrity [51]. In addition, an in vitro exposure of boar spermatozoa to ZEN or its metabolite α-zearalanol at 125 µM did not affect the acrosome integrity. However, exposure to higher concentrations (i.e., 187.5 and 250 µM) of ZEN or α-zearalenol decreased the proportion of live acrosome-reacted spermatozoa [57]. In agreement, in vitro exposure of stallion fresh semen to 0.1 mM β-zearalenol or α-zearalenol, but not to ZEN, increased the proportion of acrosome-reacted spermatozoa [58]. All together, it can be speculated that the acrosome membrane may be less sensitive to the mycotoxin’s exposure.

Likewise, the mitochondrial membrane potential is associated with mitochondrial functionality [49]; therefore, it has been suggested as a good predictor of semen quality and male fertility [59,60]. For instance, low mitochondrial membrane potential and an alteration in oxygen consumption are associated with compromised motility of spermatozoa [59,61]. The production of ROS is also associated with mitochondrial functioning. On the one hand, ROS are essential for spermatozoa capacitation and acrosome reaction [62,63]. However, in cases of ROS accumulation, they are harmful to the cell. In particular, ROS can induce oxidative stress, peroxidation of polyunsaturated fatty acids in the spermatozoa membrane, DNA fragmentation, and apoptosis [64]. For instance, a man’s reduced fertility was found to be associated with increased ROS production and lower mitochondrial membrane potential [65]. In our previous study, we found that exposure of bovine spermatozoa to 10 µM aflatoxin B1 induces an increase in the proportion of spermatozoa with high mitochondrial membrane potential, indicating hyperpolarization of the mitochondrial membrane, and it was associated with increased DNA damage [39]. Exposure to OTA at 100 µM increased ROS production in boar spermatozoa [51]. In addition, an in vitro study conducted on rabbit spermatozoa reported that exposure to T2 (1–50 µM) decreased spermatozoa motility after 8 h of incubation and that it was associated with lower mitochondrial membrane potential and membrane integrity [66]. Nevertheless, in the current study, exposure to single mycotoxins or binary mixtures did not affect the mitochondrial features. In agreement, in vitro exposure of boar spermatozoa to OTA at 10 or 100 µM for 24 h did not affect the mitochondrial membrane potential [51]. In vitro exposure of fresh bovine spermatozoa to aflatoxin B1 at 0.1, 1, or 100 µM for 2 and 4 h did not affect the mitochondrial membrane potential [39]. 

Overall, regarding the impact of mycotoxin exposure on the spermatozoa, it is possible that the descriptive effect discussed above is due to the mycotoxin level and the period that the spermatozoa were exposed. 

### 3.2. Cytotoxicity of Single Mycotoxins

Based on EFSA, mycotoxins can be divided into either regulated or unregulated groups. The regulated mycotoxins are classified as a risk to public health because of their high toxicity. Therefore, the regulations defined a maximum safe level in feed and food commodities for humans and animals regarding several mycotoxins [9,10,11]. Exposure of mouse Leydig and Sertoli cells for 24 h to OTA (0–2 µM and 0–5 µM, respectively) resulted in a significant decrease in cell proliferation in a dose-dependent manner. This was also associated with a disruption in calcium homeostasis and an alteration in the MAPK (ERK1/2 and JNK) pathways [67]. In vitro exposure of boar spermatozoa to OTA in the range of 10–200 µM for 24 h decreased spermatozoa motility and increased ROS production and apoptosis [51]. Oral administration of OTA to male mice for 45 days at 1 µg/kg body weight/day decreased the sperm count [68]; however, at 50 µg/animal/day for 46 days, it decreased the spermatozoa count in the epididymis and reduced the spermatozoa motility, viability, and fertility rate [69]. Interestingly, in the current study, no effect was observed following exposure of spermatozoa to OTA; this is most likely due to the relatively low concentrations that were used and the exposure duration. The different effects of OTA exposure might also be related to differences between species and targeted cells. Another possible explanation is that cattle are less sensitive to OTA relative to other species. A previous study in cattle indicated that the lethal dose of OTA is about 13 mg/kg body weight, whereas for goats, it is 3 mg/kg body weight [70]. These differences can at least partially explain why the relatively low OTA concentrations, even at the maximum level of 10 ppm, used in our study did not impair the cellular features of bovine spermatozoa. 

In our study, ZEN, at the range of 0.1–10 ppm, did not affect the cellular features of bovine spermatozoa. In support of this, in vitro exposure of fresh bovine spermatozoa to ZEN (0.01 to 0.5 mM for 1 h) did not affect the spermatozoa survival rate [52]. On the other hand, exposure of bovine frozen-thawed spermatozoa to 0.25 and 0.5 mM ZEN decreased their biological activity [52]. In addition, a recent study conducted on bull spermatozoa reported that the lowest concentration of ZEN that was found to affect their membrane stability was 0.01 mM on native spermatozoa. However, exposure of bull spermatozoa for 1 h to 0.25 mM ZEN deleteriously affects the activity of the frozen-thawed spermatozoa [71]. Co-culture of bovine spermatozoa with endometrial epithelial cells that were pre-treated with 1000 ng/mL ZEN (corresponding to 1 ppm ZEN) reduced the spermatozoa motility and progressive motility [72]. Incubation of boar spermatozoa with 30–95 µM ZEN for 4 h impaired their chromatin instability [73]. Moreover, ZEN has been shown to have deleterious effects on rodent reproductive systems and spermatozoa [16]. Studies in cattle reported that ZEN is generally bio-transformed into β-zearalenol rather than α-zearalenol, which is much more estrogenic than ZEN by itself [74]. Although not examined in the current study, it is possible that ZEN’s metabolites might induce a toxic effect rather than only ZEN by itself. Considering the limitation of the in vitro model, which bypasses the rumen metabolism, the effect of ZEN metabolites cannot be ruled out, and further examination is required. In addition, although not clear enough, the discrepancy between studies could be related to differences in ZEN concentrations and the exposure duration.

Studies in rodents provided evidence that T2 is a reproductive toxicant [22]. In vivo exposure of male mice to T2 (10 and 1 mg/kg body weight) decreased the number of live spermatozoa as well as the acrosome integrity; this is associated with a reduced testosterone concentration in the serum [75]. A decline in spermatozoa concentration and morphology was recorded in mice treated with T2 (0.5–2 mg/kg body weight). This was associated with a higher ROS production and a higher expression of pro-apoptotic genes in the testis [76]. Administering T2 (0.78–0.99 mg/kg body weight) to male rabbits for 3 days decreased the proportion of spermatozoa with progressive motility and increased the proportion of spermatozoa with abnormal morphology [77]. Here, we provide the first evidence that direct exposure of spermatozoa to T2, within the range of admitted limits for animal feed, did not affect any of the examined cellular features of bovine spermatozoa. In contrast, a previous study in bovine reported that in vitro exposure of fresh or frozen-thawed spermatozoa to 0.05 to 0.5 mM T2 decreased their biological activity and survival rate [52]. In addition, feeding bulls for 5 months with mold that contained 220–600 ng/g T2 resulted in low-quality spermatozoa, manifested by low progressive motility and poor morphology [78]. It is worth mentioning that in the latter studies, the concentration of T2 was significantly higher than that used in the current study, which might at least partially explain the discrepancy between the studies.

Similar to T2, in the current study, no effect on bovine cellular features was found following exposure to relatively low concentrations of DAS. In support of this, oral administration of 0–5 mg/kg body weight DAS to male broiler chickens did not affect their fertility ability; however, >10 mg/kg DAS decreased their fertility [79]. In contrast, administering DAS (0.5, 0.75, and 10 mg/kg/body weight) to male mice affected the spermatozoa morphology, mainly by increasing the abnormality patterns, and it was associated with a reduced spermatozoa count [80]. 

Overall, it is suggested that the effect of mycotoxins on the spermatozoa is mainly affected by the toxin concentration as well as the animal sensitivity, which is thought to differ between species. The findings of the current study provide the first evidence indicating that exposing bovine spermatozoa to a single mycotoxin at the range of permitted levels in animal feed did not affect the spermatozoa features. However, exposure to mixtures of mycotoxins, specifically ternary ones, which have been examined here, impairs some of the cellular features described below. Nevertheless, the fertility competence of the spermatozoa that were exposed to mycotoxins is not yet clear and deserves further investigation and study. 

### 3.3. Cytotoxicity of Binary Mycotoxin Mixtures

Evaluation of mycotoxin toxicology is largely based on a single mycotoxin rather than on a mycotoxin mixture. Recent studies highlight the fact that more than one mycotoxin generally exists in food [81,82]. Since mycotoxins can interact and exert a higher degree of damage relative to that of a single mycotoxin, co-exposure to the mycotoxin mixture is of high concern. Until 2016, about 116 mycotoxin combinations have been examined [28]. However, their toxicological impact on male reproductive health and spermatozoa quality is limited. 

There are three possible interactions between mycotoxins, classified as an additive (i.e., no interaction), synergistic (i.e., the interaction resulted in a greater effect), or antagonist (i.e., the interaction resulted in a lower effect) effect, relative to the effect of a single mycotoxin [28,29]. For instance, feeding rabbits with OTA combined with aflatoxin B1 (1 and 0.5 ppm, respectively) increased their mortality, compared with either OTA or aflatoxin B1 as a single toxin [83]. Importantly, here we report that exposure of spermatozoa to all binary compositions did not have any synergistic effect on the majority of the examined spermatozoa features, as expected. Few synergistic interactions are suggested between ZEN and T2, OTA and DAS, and OTA and T2 regarding the oxidation status, acrosome membrane integrity, and viability. In support, exposing fresh or frozen-thawed bovine spermatozoa to a ZEN and T2 mixture, ranging from 0.01 to 0.5 mM, decreased the spermatozoa’s biological activity and survival rate [52]. Regarding the possible interactions between OTA and T2 as well as OTA and DAS, to the best of our knowledge, we are the first to report any interaction on mammalian spermatozoa. 

In fact, our findings imply that most of the binary compositions resulted in additive interactions. Similarly, an additive interaction between ZEN and T2 has been previously reported following in vitro exposure of human granulo-monocytic hematopoietic progenitors [84]. In vitro exposure of murine ovarian granular KK-1 cells to a mixture of OTA and ZEN induced an additive effect on cell viability [85]. An in vivo study in pigs indicated that a mixture of OTA and T2 (2.5 mg/kg body weight and 8 mg/kg body weight, respectively) induces an additive effect on serum biochemical, hematological, and immunological values and that it is associated with reduced body and liver weight [86]. OTA, combined with DAS, exhibited an additive interaction in male broiler chicken body weight, along with an antagonistic effect on uric acid and cholesterol levels [87].

The higher toxic effect of OTA, along with T2, was previously reported as well. For instance, an injection of OTA and T2 (2 or 4 mg/kg and 0.5 mg/kg, respectively) to CD-1 mice on gestation day 10 resulted in a higher toxic effect on the developing fetuses, manifested by a higher incidence of malformations in the tail and the limb as well as higher prenatal mortality [88]. Several types of interactions, including additive, synergistic, or antagonistic, have been reported between OTA and ZEN regarding ROS generation in HepG2 cells in a dose-dependent manner [85]. In human HepG2 cells, both ZEN and OTA have been found to share some common mechanisms of action, such as induction of ROS generation and the p53-dependent apoptotic pathway [89]. Taken together, this study suggests the possible interaction effects of mycotoxins on bovine spermatozoa. 

### 3.4. Cytotoxicity of Ternary Mycotoxin Mixtures

Most of the published papers have examined the effect of binary mixtures; however, only a few studies have examined ternary mixtures, as performed in the current study. Here, we report that a ternary mycotoxin mixture of OTA + T2 + ZEN affected the mitochondrial function, as manifested by a reduction in the mitochondrial potential membrane, and that the OTA + T2 + ZEN mixture increased the proportion of spermatozoa with ROS. It is suggested that the T2 mycotoxin is responsible for the synergistic effect in the ternary mixture of these three mycotoxins. In support of this suggestion, a synergistic interaction was found between OTA and T2 and between ZEN and T2 (i.e., binary mixtures) regarding the oxidation status of the spermatozoa, and there was only an additive interaction between OTA and ZEN. 

In fact, all ternary mixtures seem to decrease the mitochondrial membrane potential relative to single mycotoxins. Impairment in mitochondrial functioning is associated with aberrant motility [90]. In support of this, oral administration of a ternary mixture of fumonisin B (5 mg/kg of the feed), DON (1 mg/kg of the feed), and ZEN (0.25 mg/kg of the feed) resulted in a synergistic effect on spermatozoa motility and testosterone synthesis in male rabbits. An additive effect was also reported on spermatogenesis and spermatozoa morphology [91]. Other interactions between three mycotoxins have also been reported, including OTA + fumonisin B1 + beauvericin in porcine kidney PK15 cells [92]; OTA+ ZEN+ α-zearalenol in human HepG2 cells [93] and others [31,37]; however, these interactions did not include the reported effects of ternary mixtures, as examined in this study, on bovine spermatozoa. 

Overall, the ternary mixtures used here seemed to affect the spermatozoa to a greater degree than the binary mixture, suggesting that the mycotoxin combination within the mixture plays a pivotal role in determining the toxicity effect and its intensity on the spermatozoa.

## 4. Conclusions

The current study is the first to report the possible effect of OTA, ZEN, DAS, and T2 as single mycotoxins or as a mixture on bovine spermatozoa. While exposing spermatozoa to a single mycotoxin or binary mixtures did not affect the cellular features of bovine spermatozoa, it can be speculated that direct exposure of spermatozoa to a single mycotoxin within the range of admitted limits for animal feed seems to be safe. However, considering the possible interactions, binary or ternary mixtures are considered harmful. Nonetheless, one of this model’s limitations is that it bypasses the rumen, and the effect of the mycotoxins’ metabolites cannot be ruled out. Thus, further evaluations are required to determine the impact of these mycotoxins on the fertility ability of spermatozoa, as a fundamental part of fertility success. 

## 5. Materials and Methods

All chemicals were purchased from ‘Sigma-Merck’ (Rehovot, Israel) unless otherwise indicated. All five mycotoxins (ZEN, DAS, OTA, and T2) were purchased from Fermentek (Jerusalem, Israel). The mycotoxins, ZEN, DAS, OTA, and T2, were dissolved in absolute methanol (100%) to prepare stock solutions at 1000 ppm. Working solutions were freshly prepared by serial dilution in NKM buffer (110 mM NaCl, 5 mM KCl, and 20 nM MOPS (3-N-morphilino propanesulfonic acid; pH 7.4).

### 5.1. Mycotoxin Concentration

Since there is a lack of information regarding the actual levels of mycotoxins in testicular tissue, the chosen concentrations in this study were based on the Animal Feed Inspection Division requirements for the issue/renewal of a permit to manufacture, market, and trade in animal feed, which is based on the Israeli Control of Animal Feed law, 2014, along with the recommendation of the EU Commission Recommendation and the European Food Safety Authority [9,10,11]. Accordingly, the examined concentrations of each mycotoxin are listed in Table 6.

### 5.2. Sample Preparation

The experiments were conducted using frozen-thawed semen from 6 mature working bulls (6.0 ± 1.3 years old) from SION—Israeli Company for Artificial Insemination and Breeding Ltd.—which participates in the breeding program of the Israeli Cattle Breeders Association. Semen was collected in a disposable tube using a sterile heated (38 °C) artificial vagina, and it was immediately transferred to the laboratory and subjected to the cryopreservation procedure routinely conducted at SION [94]. Briefly, collected ejaculated spermatozoa were diluted (1:10 *v*/*v* at room temperature) with extender [10% (*v*/*v*) glycerol, 20% (*w*/*v*) egg yolk, 20 mg lactose, 1000 IU penicillin, and 500 mg streptomycin]. Then, the samples were chilled for 3 h to 4 °C, and about 21.92 ± 2.53 × 10^6^ spermatozoa were inserted into 0.25 mL chilled straws. Straws were cooled for 10 min to −95 °C in a programmed box with a vapor nitrogen-saturated atmosphere and then plunged into liquid nitrogen until further analysis.

#### Mycotoxin Exposure

Each sample, consisting of two straws from the same bull, was thawed in a prewarmed bath at 38.5 °C for 1 min. Then, samples were subjected to the ‘swim up’ procedure in which they were washed in NKM buffer (38.5 °C, pH 7.4) and centrifuged at 600× *g* for 10 min at room temperature. Then, samples were incubated for 20 min at 38.5 °C to enable the live-motile spermatozoa to swim up. Then 100 µL from each sample (i.e., each bull) was transferred into a new experimental tube containing 500 µL NKM buffer with (1) methanol (4% *v*/*v*); (2) a single mycotoxin (ZEN, OTA, DAS, or T2); (3) binary mixtures (DAS + T2, OTA + DAS, OTA + T2, OTA + ZEN, ZEN + T2, or ZEN + DAS); (4) ternary mixtures (OTA + DAS + T2, OTA + ZEN + T2, or ZEN + DAS + T2); (5) without any supplement (the untreated group). 

Each sample was equally divided into the experimental tubes, including adequate amounts of mycotoxin solution or solvent. For the single mycotoxin exposure, the concentrations used are listed in Table 1. For each binary combination, three concentrations (the lowest, intermediate, and highest) were chosen for each mycotoxin (ZEN: 0.1, 1, 10 ppm; OTA: 0.05, 0.25, 10 ppm; T2: 0.01, 0.1, 0.5 ppm, and DAS: 0.01, 0.1, 0.5 ppm). For each ternary combination, the intermediate concentration for each mycotoxin was used (ZEN: 1 ppm; OTA: 0.25 ppm; T2: 0.1 ppm, and DAS: 0.1 ppm). Samples were in-vitro-cultured for 2 h in a warm bath at 38.5 °C (i.e., the average core body temperature of cattle) and then subjected to the cell-based assessment of spermatozoa. 

### 5.3. Cell-Based Assessment of Spermatozoa

Spermatozoa were evaluated using a Guava EasyCyte microcapillary flow cytometer with CytoSoft software v 3.0 (Guava Technologies, Inc., Hayward, CA, USA) and ready-to-use flow cytometry kits containing lyophilized fluorochromes in each well (IMV Technologies, L’Aigle, France) as previously reported [95,96]. Flow cytometry tests were performed on frozen samples using a Guava EasyCyte microcapillary flow cytometer with CytoSoft software (Guava Technologies; distributed by IMV Technologies). This device detects particle emission properties with three photomultiplier tubes (green: 525/30 nm, yellow: 583/26 nm, and red: 655/50 nm) as well as the accompanying optical filters and splitters. Assessment included plasma membrane integrity, acrosomal membrane integrity, and mitochondrial features, including mitochondrial membrane potential and oxidation status, i.e., the production of ROS. A signal from 5000 spermatozoa was counted for each sample within each examined parameter.

Calibration was performed using the EasyCyte Check Kit (ref. 023066; IMV Technologies, L’Aigle, France) according to the manufacturer’s instructions. Briefly, 10 µL of EasyCheck reagent beads was diluted in 190 µL of EasyCheck diluent, mixed thoroughly, and run through the instrument in three replicates. The obtained results were compared to the intensity information attached to the kit for green and red lasers. Only runs with adjusted values for all lasers were used for further analysis [96].

#### 5.3.1. Plasma Membrane Integrity 

Plasma membrane integrity was evaluated using the EasyKit 1 Viability and Concentration (ref. 024708; IMV Technologies) according to the manufacturer’s instructions and as previously reported [95,96]. The kit contains two fluorochromes and allows one to distinguish between live and dead spermatozoa. From each sample, 2 µL of homogeneous spermatozoa at 57 × 106/mL was added to the well of a 96-well plate containing 199 µL EasyBuffer B (ref. 023826; IMV Technologies). The contents of each well were homogenized by pipetting, and the plate was covered and placed in an oven at 38.5 °C and protected from light for 10 min. A total of 5000 spermatozoa were counted in the flow cytometry reading. The results are expressed as the percentage of viable spermatozoa. 

#### 5.3.2. Acrosome Membrane Integrity

The acrosome membrane integrity was evaluated with EasyKit 5 (ref. 025293; IMV Technologies) according to the manufacturer’s instructions with minor modifications, as previously described [95,96]. The kit enables one to distinguish between spermatozoa with intact (intense green fluorescence), reacted (a low fluorescence signal), or damaged acrosome (no fluorescence). A volume of 2 µL of homogeneous spermatozoa at 57 × 106/mL was added to each well of a 96-well plate containing 199 µL EasyBuffer B (ref. 023826; IMV Technologies). The plate was covered to protect the samples from light and placed in an oven at 38.5 °C for 45 min. Then, the plate was loaded into the flow cytometer for signal reading. A signal from 5000 spermatozoa was counted, and the results were expressed as the spermatozoa percentage with an intact or damaged acrosomal membrane. 

#### 5.3.3. Mitochondrial Features

##### Mitochondrial Membrane Potential 

The mitochondrial membrane potential of the spermatozoa was evaluated using EasyKit 2 (ref. 024864; IMV Technologies) according to the manufacturer’s instructions and as previously reported [95,96]. The kit allows one to distinguish between spermatozoa with either polarized (i.e., high mitochondrial membrane potential) that appears as orange fluorescence or depolarized (i.e., low mitochondrial membrane potential) that appears as green fluorescence. A volume of 10 µL absolute ethanol was added to each well to suspend the fluorochrome, followed by the addition of 190 µL phosphate-buffered saline (PBS). Then, 2 µL of homogeneous spermatozoa at 57 × 106/mL from each sample was added separately to the wells of a 96-well plate. The plate was covered to protect the samples from light and placed in an oven at 38.5 °C for 30 min. A total of 5000 spermatozoa were counted. The ratio between the percentages of spermatozoa expressing polarized and depolarized mitochondrial membranes was calculated and log-transformed. 

##### Oxidation Status

The level of ROS was evaluated with EasyKit 3 (ref. 025157; IMV Technologies) according to the manufacturer’s instructions and as previously described [45,96]. The kit allows one to distinguish between live and dead spermatozoa with either an intense fluorescence mean of oxidated cells (i.e., ROS+) or not (i.e., ROS-). A volume of 2 µL of homogeneous sperm at 57 × 106/mL was added to each well of a 96-well plate containing 199 µL pre-warmed PBS (38.5 °C). The plate was covered to protect the samples from light and placed in an oven at 38.5 °C for 20 min. Then, 2 µL of 39 mM hydrogen peroxide was added to each well and incubated for an additional 40 min at 38.5 °C. Thereafter, spermatozoa were washed with 600 µL prewarmed PBS at 38.5 °C and centrifuged for 5 min at 300× *g*. The pellet was suspended in 200 µL PBS, placed in a 96-well plate, and loaded into the flow cytometer. A signal from 5000 spermatozoa was counted, and the results were expressed as the percentage of viable spermatozoa with ROS (i.e., ROS+-spermatozoa). 

### 5.4. Estimation of the Interactive Effects of Combined Mycotoxins

The effect of interactions between mycotoxins was interpreted by comparing the measured and expected values, as previously reported [31,97], but with some modifications. Prior to the comparison, all values from all examined parameters from single, binary, or ternary mixtures were adjusted relative to the control (i.e., with solvent) in order to eliminate the solvent effect. The expected values were obtained by the following formula:

(1) For the binary mixtures:
  a. Mean expected _adjusted to control_ (mycotoxin 1+ mycotoxin 2) = mean _adjusted to control_ (mycotoxin 1) + mean _adjusted to control_ (mycotoxin 2)
  b. SEM expected (mycotoxin 1 + mycotoxin 2) = [(SEM for mycotoxin 1)^2^ + (SEM for mycotoxin 2)^2^]^1/2^
(2) For the ternary mixtures:
  a. Mean expected adjusted to control (mycotoxin 1+ mycotoxin 2+ mycotoxin 3) = mean adjusted to control (mycotoxin 1) + mean adjusted to control (mycotoxin 2) + mean adjusted to control (mycotoxin 3)
  b. SEM (expected for mycotoxin 1 + mycotoxin 2 + mycotoxin 3) = [(SEM for mycotoxin 1)2 + (SEM for mycotoxin 2)2 + (SEM for mycotoxin 3)2]_1/2_

The expected values of the viable spermatozoa, the proportion of spermatozoa with a damaged or intact acrosome membrane, the log(ratio of spermatozoa expressing polarized vs. depolarized mitochondrial membranes), and the proportion of ROS+-spermatozoa were calculated and compared to their measured values, respectively. Statistical comparisons were conducted using appropriate *t*-tests (*t* = (measured-a)/(b)) in each case. The obtained *p*-values were used to interpret the mixture’s effect as follows: (1) a significant difference between the measured and the expected effect, i.e., the measured effect was higher than the expected effect was interpreted as a synergistic interaction. (2) A significant difference between the measured and the expected effect, i.e., the measured effect was lower than the expected effect and was interpreted as an antagonistic interaction. (3) No significant difference between the measured and the expected effect was interpreted as an additive interaction. 

### 5.5. Statistical Analysis

Data were analyzed using JMP software v 16.0 (SAS Institute, Inc., 2004, Cary, NC, USA). Differences between groups were statistically analyzed using ANOVA, followed by Dunnett’s (i.e., relative to the control) method for multiple comparisons. Variables included the proportion of viable spermatozoa, the proportion of spermatozoa with a damaged or intact acrosome membrane, the log(ratio of spermatozoa expressing polarized vs. depolarized mitochondrial membranes), and the proportion of ROS+-spermatozoa. Data are presented as the mean ± SEM. *p* < 0.05 was considered significant.

## Figures and Tables

**Table 1:** Effect of a mycotoxin solvent, methanol, on spermatozoa’s cellular features relative to an untreated control.

Group	* Viability (%)	*p*-Value	** Mitochondrial Membrane Potential Log(Ratio)	*p*-Value	* Intact Acrosome Membrane (%)	*p*-Value	* Viable Spermatozoa with ROS (%)	*p*-Value
Untreated	43.71 ± 2.7	0.98	0.17 ± 0.2	0.25	56.35 ± 1.9	0.89	6.94 ± 1.2	0.32
Control	43.64 ± 3.5		0.53 ± 0.2		56.73 ± 2.0		8.67 ± 1.2	

ROS: Reactive oxygen species. * Data are presented as the mean ± SEM. ** Data are presented as the mean of the ratio of spermatozoa polarized relative to those with a depolarized mitochondrial membrane.

**Table 2:** Dose-response study to examine the effect of mycotoxins on the cellular features of bovine spermatozoa.

Mycotoxin	Concentration (ppm)	* Viability (%)	** Mitochondrial Membrane Potential	* Acrosome Membrane Integrity (%)		* Oxidation Status of Viable Spermatozoa (%)	
				Damage	Intact	ROS-	ROS+
DAS	0 (control)	37.9 ± 8.5	0.23 ± 0.42	6.9 ± 0.5	59.9 ± 1.9	53.4 ± 10.1	9.4 ± 2.4
	0.01	44.3 ± 4.9	−0.07 ± 0.42	6.5 ± 2.4	59.2 ± 2.4	53.5 ± 6.1	8.9 ± 1.6
	0.05	44.9 ± 4.1	−0.25 ± 0.34	5.0 ± 1.4	62.0 ± 1.2	55.4 ± 10.0	7.7 ± 2.1
	0.1	44.8 ± 4.3	0.36 ± 0.48	4.9 ± 1.4	61.0 ± 1.6	45.3 ± 7.2	8.8 ± 1.5
	0.5	44.0 ± 5.2	−0.02 ± 0.39	7.9 ± 2.1	56.7 ± 2.6	47.3 ± 7.1	7.2 ± 1.9
OTA	0 (control)	47.9 ± 5.1	0.32 ± 0.34	5.9 ± 1.4	52.4 ± 5.1	66.3 ± 5.1	3.9 ± 0.9
	0.05	47.2 ± 5.1	0.07 ± 0.33	6.6 ± 2.6	54.9 ± 5.0	65.9 ± 5.2	4.1 ± 1.1
	0.1	44.3 ± 4.7	0.24 ± 0.38	5.1 ± 1.0	57.1 ± 5.3	59.9 ± 7.2	3.8 ± 1.2
	0.25	43.1 ± 3.5	0.33 ± 0.49	6.9 ± 2.6	53.6 ± 5.4	63.0 ± 5.5	4.8 ± 1.0
	1	44.5 ± 3.2	0.46 ± 0.36	4.4 ± 1.7	55.1 ± 5.8	62.3 ± 2.5	5.5 ± 1.6
	10	44.8 ± 5.2	0.18 ± 029	6.4 ± 1.1	55.4 ± 5.9	62.3 ± 3.9	3.4 ± 0.8
T2	0 (control)	38.5 ± 9.1	−0.04 ± 0.35	9.2 ± 2.3	59.2 ± 6.5	61.7 ± 8.4	7.4 ± 1.5
	0.01	41.5 ± 7.1	0.15 ± 0.28	7.9 ± 2.0	58.3 ± 5.3	63.2 ± 8.1	5.4 ± 1.2
	0.05	41.2 ± 7.7	0.31 ± 0.56	9.0 ± 2.5	58.2 ± 6.1	60.6 ± 6.2	6.4 ± 1.1
	0.1	38.9 ± 6.4	0.42 ± 0.44	8.5 ± 2.2	58.7 ± 5.7	61.6 ± 7.7	6.6 ± 2.1
	0.5	45.3 ± 7.0	0.19 ± 0.32	10.2 ± 2.0	52.9 ± 5.9	56.2 ± 7.7	11.4 ± 4.2
ZEN	0 (control)	49.2 ± 5.4	1.53 ± 0.53	3.0 ± 0.5	55.7 ± 2.3	29.0 ± 1.1	7.1 ± 1.4
	0.1	47.1 ± 4.7	0.87 ± 0.26	3.1 ± 0.4	57.3 ± 2.1	24.8 ± 3.4	8.7 ± 1.6
	0.5	43.6 ± 4.9	1.05 ± 0.52	4.6 ± 1.3	54.6 ± 2.2	24.8 ± 1.3	8.4 ± 0.6
	1	48.2 ± 5.2	1.36 ± 0.53	3.3 ± 0.4	55.8 ± 2.8	25.2 ± 2.5	8.2 ± 2.1
	5	46.5 ± 5.3	0.37 ± 0.26	4.1 ± 1.0	55.4 ± 2.5	26.6 ± 1.6	10.4 ± 0.6
	10	48.2 ± 3.5	0.71 ± 0.32	5.1 ± 1.3	53.6 ± 2.2	33.5 ± 1.5	3.3 ± 0.6

ROS: Reactive oxygen species, “ROS-“ or “ROS+” refers to spermatosoa without or with expression of ROS, respectively; ZEN: zearalenone; OTA: ochratoxin; T2: T-2 toxin; DAS: Diacetoxyscirpenol; control (i.e., with solvent). * Data are presented as the mean ± SEM. ** Data are presented as the mean log(mean ratio) ± SEM.

**Table 3:** The effect of binary combinations of mycotoxins on the cellular features of bovine spermatozoa.

Mixture	* Mixture	** Viability (%)	*** Mitochondrial Membrane Potential	** Acrosomal Membrane Integrity (%)		** Oxidation Status of Viable Spermatozoa (%)	
				Damage	Intact	ROS-	ROS+
DAS + T2	Control	46.0 ± 6.8	0.42 ± 0.5	3.2 ± 1.5	64.4 ± 6.9	35.2 ± 2.4	8.0 ± 2.8
	Low	53.8 ± 4.5	0.29 ± 0.3	3.0 ± 0.8	64.3 ± 0.9	32.9 ± 1.5	10.1 ± 2.3
	Intermediate	43.1 ± 9.2	−0.29 ± 0.3	3.5 ± 2.2	67.0 ± 3.7	34.4 ± 5.5	11.1 ± 2.6
	High	43.9 ± 9.3	0.25 ± 0.3	5.1 ± 1.7	62.9 ± 3.4	30.7 ± 3.2	10.4 ± 3.4
OTA + DAS	Control	56.8 ± 8.4	0.58 ± 0.2	4.5 ± 1.0	63.8 ± 6.7	38.6 ± 10.8	18.9 ± 1.5
	Low	60.3 ± 7.9	0.51 ± 0.1	3.5 ± 1.4	66.5 ± 8.5	36.2 ± 10.2	16.9 ± 2.4
	Intermediate	56.3 ± 7.3	0.85 ± 0.2	4.1 ± 1.4	64.9 ± 7.7	29.5 ± 7.6	20.03 ± 1.2
	High	56.3 ± 7.6	0.49 ± 0.4	3.2 ± 0.8	66.1 ± 7.4	38.7 ± 9.9	14.9 ± 4.0
OTA + T2	Control	37.2 ± 10.8	0.89 ± 0.2	11.2 ± 3.8	54.8 ± 2.6	37.8 ± 4.7	17.1 ± 2.4
	Low	37.8 ± 11.8	1.26 ± 0.1	6.4 ± 1.2	59.7 ± 3.8	39.7 ± 4.8	16.7 ± 3.0
	Intermediate	37.2 ± 10.8	1.21 ± 0.1	7.6 ± 3.8	58.5 ± 4.5	31.7 ± 5.8	16.6 ± 3.4
	High	39.1 ± 11.5	1.05 ± 0.1	9.9 ± 5.6	58.5 ± 7.5	35.01 ± 4.5	20.5 ± 2.9
OTA + ZEN	Control	42.3 ± 13.7	0.69 ± 0.3	9.4 ± 3.5	69.2 ± 0.8	14.8 ± 4.7	44.1 ± 8.0
	Low	38.7 ± 12.2	0.93 ± 0.9	8.2 ± 4.1	70.1 ± 4.2	15.6 ± 5.0	42.1 ± 8.2
	Intermediate	37.9 ± 12.9	0.69 ± 0.4	8.6 ± 2.0	72.4 ± 2.1	13.3 ± 5.1	41.9 ± 9.7
	High	38.8 ± 8.6	0.66 ± 0.3	11.6 ± 2.6	67.1 ± 3.5	11.8 ± 3.9	45.3 ± 9.8
ZEN + T2	Control	57.1 ± 11.7	2.09 ± 0.6	4.8 ± 0.9	69.8 ± 1.8	30.6 ± 8.6	19.0 ± 0.3
	Low	55.3 ± 7.0	0.87 ± 0.1	3.4 ± 0.5	70.9 ± 2.3	35.2 ± 6.9	15.2 ± 1.6
	Intermediate	57.3 ± 8.8	1.08 ± 0.1	4.2 ± 1.8	73.5 ± 2.1	36.7 ± 11.1	12.7 ± 4.2
	High	56.3 ± 7.6	0.79 ± 0.2	2.8 ± 0.5	74.0 ± 3.5	34.2 ± 7.8	17.4 ± 0.3
ZEN + DAS	Control	51.3 ± 11.0	0.81 ± 0.9	3.0 ± 1.7	62.7 ± 4.0	65.6 ± 4.3	7.8 ± 2.4
	Low	57.3 ± 8.4	0.36 ± 0.9	3.1 ± 0.8	60.4 ± 6.7	53.8 ± 5.4	8.6 ± 0.9
	Intermediate	49.7 ± 9.7	1.04 ± 0.3	2.3 ± 0.6	61.5 ± 5.6	47.6 ± 8.9	10.0 ± 0.9
	High	54.1 ± 7.2	0.06 ± 0.8	3.4 ± 0.5	60.9 ± 4.5	50.0 ± 12.5	9.7 ± 2.4

ROS: Reactive oxygen species; ZEN: zearalenone; OTA: ochratoxin; T2: T-2 toxin; DAS: Diacetoxyscirpenol. ROS: Reactive oxygen species, “ROS-“ or “ROS+” refers to spermatosoa without or with expression of ROS, respectively; ZEN: zearalenone; OTA: ochratoxin; T2: T-2 toxin; DAS: Diacetoxyscirpenol; control (i.e., with solvent). * Mixtures are based on the chosen concentrations from each mycotoxin; low, intermediate, and highest (ZEN: 0.1, 1, 10 ppm; OTA: 0.05, 0.25, 10 ppm; T2: 0.01, 0.1, 0.5 ppm and DAS: 0.01, 0.1, 0.5 ppm) and control group (i.e., without mycotoxin). ** Data are presented as the mean ± SEM. *** Data are presented as the log(mean ratio) ± SEM.

**Table 4:** (**a**) Suggested interactions in a binary mixture of DAS + T2. (**b**) Suggested interactions in a binary mixture of OTA + DAS. (**c**) Suggested interactions in a binary mixture of OTA + T2. (**d**) Suggested interactions in a binary mixture of OTA + ZEN. (**e**) Suggested interactions in a binary mixture of T2 + ZEN. (**f**) Suggested interactions in a binary mixture of ZEN + DAS.

(**a**)						
**Mixture**	**Examined** **Parameter**	**Concentration Combination**	**Expected Value**	**Measured Value**	***p*** **-Value**	**Interaction**
DAS + T2	Viable	Low	−5.7 ± 4.68	7.80 ± 5.28	0.1	Additive
		Intermediate	−6.81 ± 5.57	−2.84 ± 3.63	0.57	Additive
		High	−6.67 ± 6.05	−2.14 ± 3.42	0.54	Additive
	ΔΨm	Low	−1.48 ± 1.36	−0.13 ± 0.12	0.36	Additive
		Intermediate	−1.24 ± 1.08	−0.71 ± 0.22	0.65	Additive
		High	−1.45 ± 1.19	−0.17 ± 0.58	0.37	Additive
	Damaged	Low	−2.88 ± 1.68	−0.21 ± 0.86	0.21	Additive
	acrosome	Intermediate	−1.73 ± 1.22	0.29 ± 0.95	0.24	Additive
		High	−0.96 ± 3.26	1.61 ± 1.37	0.45	Additive
	Intact	Low	2.47 ± 1.08	−0.08 ± 6.58	0.71	Additive
	acrosome	Intermediate	0.83 ± 4.09	2.51 ± 4.00	0.71	Additive
		High	−4.16 ± 6.62	−1.56 ± 5.42	0.72	Additive
	Viable	Low	−8.69 ± 7.04	−2.29 ± 1.55	0.41	Additive
	ROS-	Intermediate	−7.44 ± 10.76	−0.83 ± 3.14	0.58	Additive
		High	−4.55 ± 10.22	−4.49 ± 1.13	0.99	Additive
	Viable	Low	0.49 ± 2.49	2.14 ± 1.32	0.58	Additive
	ROS+	Intermediate	−3.72 ± 3.54	3.13 ± 0.39	0.1	Additive
		High	−4.96 ± 2.29	2.47 ± 2.11	0.1	Additive
(**b**)						
**Mixture**	**Examined** **Parameter**	**Concentration Combination**	**Expected Value**	**Measured Value**	***p*** **-Value**	**Interaction**
OTA + DAS	Viable	Low	−3.61 ± 5.03	−0.50 ± 1.55	0.51	Additive
		Intermediate	−11.77 ± 3.39	−0.48 ± 1.51 *	0.02	Synergistic
		High	−4.60 ± 6.79	3.49 ± 0.81	0.21	Additive
	ΔΨm	Low	−1.22 ± 0.96	−0.07 ± 0.23	0.31	Additive
		Intermediate	−0.22 ± 0.67	0.27 ± 0.46	0.64	Additive
		High	−1.06 ± 0.90	−0.09 ± 0.67	0.51	Additive
	Damaged	Low	0.74 ± 1.09	−0.40 ± 0.85	0.32	Additive
	acrosome	Intermediate	2.92 ± 2.99	−1.25 ± 0.87	0.22	Additive
		High	2.93 ± 0.84	−0.96 ± 0.51 *	0.007	Antagonist
	Intact	Low	0.26 ± 1.17	1.08 ± 2.14	0.78	Additive
	acrosome	Intermediate	−2.32 ± 4.01	2.25 ± 1.42	0.3	Additive
		High	−7.19 ± 3.51	2.71 ± 2.00 *	0.05	Synergistic
	Viable	Low	−1.76 ± 3.47	−2.37 ± 1.76	0.77	Additive
	ROS-	Intermediate	−5.16 ± 9.29	−9.11 ± 4.45	0.7	Additive
		High	−6.64 ± 10.81	0.15 ± 1.15	0.47	Additive
	Viable	Low	0.22 ± 2.40	−2.00 ± 1.41	0.44	Additive
	ROS+	Intermediate	−2.06 ± 3.36	1.11 ± 1.48	0.44	Additive
		High	−3.66 ± 3.19	−4.01 ± 3.25	0.97	Additive
(**c**)						
**Mixture**	**Examined** **Parameter**	**Concentration Combination**	**Expected Value**	**Measured Value**	***p*** **-Value**	**Interaction**
OTA + T2	Viable	Low	−1.04 ± 3.04	0.58 ± 1.26	0.65	Additive
		Intermediate	−10.98 ± 4.59	−0.08 ± 0.69 *	0.05	Synergistic
		High	−4.55 ± 4.34	1.86 ± 1.10	0.2	Additive
	ΔΨm	Low	−0.69 ± 0.99	0.37 ± 0.44	0.36	Additive
		Intermediate	−0.21 ± 0.91	0.32 ± 0.37	0.61	Additive
		High	−0.63 ± 1.07	0.16 ± 0.31	0.51	Additive
	Damaged	Low	−2.65 ± 1.97	−4.87 ± 3.66	0.61	Additive
	acrosome	Intermediate	0.98 ± 2.99	−3.64 ± 6.31	0.54	Additive
		High	−1.78 ± 3.25	−1.38 ± 8.70	0.97	Additive
	Intact	Low	3.78 ± 1.57	4.87 ± 3.84	0.8	Additive
	acrosome	Intermediate	1.16 ± 1.42	3.71 ± 6.86	0.75	Additive
		High	1.11 ± 2.98	3.63 ± 9.77	0.81	Additive
	Viable	Low	−5.19 ± 6.66	1.93 ± 3.26	0.37	Additive
	ROS-	Intermediate	−5.39 ± 7.24	−6.08 ± 2.00	0.93	Additive
		High	−8.91 ± 5.85	−2.81 ± 0.31	0.33	Additive
	Viable	Low	−1.17 ± 1.05	−0.44 ± 3.38	0.84	Additive
	ROS+	Intermediate	−2.91 ± 1.61	−0.55 ± 1.47	0.32	Additive
		High	−1.54 ± 1.09	3.36 ± 2.07	0.08	Additive
(**d**)						
**Mixture**	**Examined** **Parameter**	**Concentration Combination**	**Expected Value**	**Measured Value**	***p*** **-Value**	**Interaction**
OTA + ZEN	Viable	Low	−1.93 ± 2.66	−3.64 ± 2.78	0.74	Additive
		Intermediate	−8.74 ± 3.83	−4.46 ± 1.72	0.38	Additive
		High	−2.69 ± 7.44	−3.56 ± 5.21	0.86	Additive
	ΔΨm	Low	−0.74 ± 0.74	0.24 ± 0.77	0.38	Additive
		Intermediate	0.67 ± 0.25	0.001 ± 0.47	0.3	Additive
		High	−0.85 ± 0.84	−0.02 ± 0.26	0.37	Additive
	Damaged	Low	0.63 ± 1.79	−1.16 ± 1.96	0.47	Additive
	acrosome	Intermediate	4.33 ± 1.44	−0.77 ± 3.19	0.39	Additive
		High	2.13 ± 3.65	2.21 ± 4.08	0.99	Additive
	Intact	Low	3.26 ± 1.15	0.96 ± 3.68	0.65	Additive
	acrosome	Intermediate	−4.07 ± 1.42	3.23 ± 2.64	0.25	Additive
		High	−1.98 ± 3.04	−2.11 ± 3.67	0.98	Additive
	Viable	Low	−2.48 ± 9.06	0.82 ± 0.49	0.58	Additive
	ROS-	Intermediate	−6.27 ± 8.17	−1.55 ± 1.08	0.58	Additive
		High	−1.93 ± 5.22	−2.97 ± 1.67	0.86	Additive
	Viable	Low	1.06 ± 2.99	−2.01 ± 0.36	0.37	Additive
	ROS+	Intermediate	0.84 ± 4.66	−1.55 ± 2.57	0.62	Additive
		High	−3.95 ± 1.80	1.18 ± 2.96	0.18	Additive
(**e**)						
**Mixture**	**Examined** **Parameter**	**Concentration** **Combination**	**Expected Value**	**Measured Value**	***p*** **-Value**	**Interaction**
T2 + ZEN	Viable	Low	−3.86 ± 1.92	0.19 ± 2.98	0.29	Additive
		Intermediate	−3.19 ± 5.85	−0.83 ± 4.3	0.76	Additive
		High	−2.54 ± 6.76	−1.82 ± 6.48	0.94	Additive
	ΔΨm	Low	−1.08 ± 1.21	−1.23 ± 0.78	0.92	Additive
		Intermediate	−0.49 ± 0.88	−1.01 ± 0.49	0.62	Additive
		High	−1.45 ± 1.14	−1.31 ± 0.78	0.92	Additive
	Damaged	Low	−3.37 ± 1.79	−0.54 ± 0.94	0.21	Additive
	acrosome	Intermediate	−1.43 ± 1.44	−1.97 ± 1.48	0.8	Additive
		High	−1.67 ± 3.65	−1.39 ± 1.42	0.94	Additive
	Intact	Low	4.93 ± 1.15	3.69 ± 1.26	0.49	Additive
	acrosome	Intermediate	0.63 ± 1.42	4.19 ± 2.43	0.25	Additive
		High	0.58 ± 3.04	1.03 ± 1.24	0.89	Additive
	Viable	Low	−10.56 ± 9.06	−11.76 ± 6.62	0.92	Additive
	ROS-	Intermediate	−7.66 ± 8.17	−18.01 ± 8.61	0.42	Additive
		High	1.93 ± 5.22	−15.60 ± 13.06	0.26	Additive
	Viable	Low	1.08 ± 2.98	0.81 ± 2.91	0.5	Additive
	ROS+	Intermediate	−1.28 ± 4.66	2.14 ± 1.73	0.52	Additive
		High	−5.18 ± 1.80	1.87 ± 1.86 *	0.03	Synergistic
(**f**)						
**Mixture**	**Examined** **Parameter**	**Concentration Combination**	**Expected Value**	**Measured Value**	***p*** **-Value**	**Interaction**
ZEN + DAS	Viable	Low	−6.99 ± 4.43	−1.60 ± 2.78	0.34	Additive
		Intermediate	−4.28 ± 4.96	2.82 ± 4.00	0.31	Additive
		High	−4.04 ± 8.55	6.03 ± 5.73	0.36	Additive
	ΔΨm	Low	−1.57 ± 1.19	−0.45 ± 0.12	0.39	Additive
		Intermediate	−0.41 ± 0.63	0.22 ± 0.64	0.5	Additive
		High	−1.70 ± 0.98	−0.75 ± 0.49	0.42	Additive
	Damaged	Low	0.38 ± 0.73	−0.72 ± 1.09	0.43	Additive
	acrosome	Intermediate	0.58 ± 0.71	0.37 ± 1.76	0.92	Additive
		High	3.10 ± 1.86	0.09 ± 1.49	0.25	Additive
	Intact	Low	1.52 ± 0.47	−1.18 ± 1.62	0.16	Additive
	acrosome	Intermediate	−3.07 ± 1.95	−1.77 ± 2.25	0.68	Additive
		High	−7.47 ± 3.55	−2.27 ± 3.55	0.34	Additive
	Viable	Low	−6.61 ± 7.06	4.62 ± 1.79	0.17	Additive
	ROS-	Intermediate	−7.28 ± 10.03	6.17 ± 4.26	0.26	Additive
		High	2.54 ± 10.48	3.58 ± 4.72	0.93	Additive
	Viable	Low	2.52 ± 3.69	−3.86 ± 1.31	0.15	Additive
	ROS+	Intermediate	−0.28 ± 5.51	−6.33 ± 4.48	0.43	Additive
		High	−7.45 ± 3.51	−1.63 ± 3.24	0.27	Additive

ROS: Reactive oxygen species, “ROS-“ or “ROS+” refers to spermatosoa without or with expression of ROS, respectively; ZEN: zearalenone; OTA: ochratoxin; T2: T-2 toxin; DAS: Diacetoxyscirpenol. Examined parameters included Viable—the proportion of viable spermatozoa; ΔΨm—the ratio between the proportion of spermatozoa with polarized to depolarized mitochondrial membrane (log-transformed); Damaged and Intact acrosome—the proportion of spermatozoa with damaged or intact acrosome membrane integrity; Viable ROS- and ROS+—the proportion of viable spermatozoa without ROS or with ROS, respectively. Mixtures were made with the low, intermediate, and highest concentrations: ZEN (0.1, 1, and 10 ppm); OTA (0.05, 0.25, and 10 ppm); T2 (0.01, 0.1, and 0.5 ppm) and DAS (0.01, 0.1, and 0.5 ppm), respectively. The ‘Measured’ value is the actual outcome of each examined parameter obtained following exposure of spermatozoa to the binary mixtures, and the ‘Expected’ value was calculated for each examined parameter as the SUM of the effect obtained for two single mycotoxins. Presented are the values of the means ± S.E.M., which were adjusted relative to the control (i.e., with solvent). * *p* ≤ 0.05. Three interaction effects are suggested: (1) a synergistic interaction when the measured value was significantly higher relative to the expected value, (2) an antagonistic interaction when the measured value was significantly lower relative to the expected value, and (3) an additive interaction when the expected value was similar to that of the measured value (i.e., not significant).

**Table 5:** Suggested interactions between three mycotoxins at the intermediate concentrations.

Mixture	Examined Parameter	Expected Value	Measured Value	*p*-Value	Interaction
OTA + T2 + ZEN	Viable	−11.31 ± 5.92	−0.83 ± 2.01	0.13	Additive
	ΔΨm	−0.05 ± 0.91	−1.42 ± −3.54	0.22	Additive
	Damaged acrosome	1.43 ± 3.32	−2.86 ± 0.87	0.25	Additive
	Intact acrosome	−0.48 ± 3.96	3.17 ± 1.98	0.43	Additive
	Viable ROS-	−9.14 ± 8.84	−4.40 ± 8.11	0.7	Additive
	Viable ROS+	−1.83 ± 4.73	18.35 ± 9.01	0.08	Additive
DAS + T2 + OTA	Viable	−14.93 ± 5.64	1.87 ± 2.47 *	0.02	Synergistic
	ΔΨm	−0.79 ± 1.10	−0.97 ± 0.35	0.88	Additive
	Damaged acrosome	1.12 ± 3.23	−3.47 ± 0.90	0.21	Additive
	Intact acrosome	−0.27 ± 4.08	4.09 ± 0.81	0.33	Additive
	Viable ROS-	−8.92 ± 11.28	−2.48 ± 5.43	0.32	Additive
	Viable ROS+	−4.26 ± 3.63	2.16 ± 4.63	0.31	Additive
DAS + T2 + ZEN	Viable	−7.14 ± 6.70	5.97 ± 2.92	0.11	Additive
	ΔΨm	−1.07 ± 1.08	−0.63 ± 0.69	0.74	Additive
	Damaged acrosome	−1.29 ± 1.46	0.44 ± 0.80	0.97	Additive
	Intact acrosome	−0.81 ± 2.09	0.61 ± 0.28	0.52	Additive
	Viable ROS-	−11.19 ± 11.89	−3.17 ± 9.75	0.62	Additive
	Viable ROS+	−2.64 ± 5.68	1.07 ± 4.45	0.69	Additive

ROS: Reactive oxygen species, “ROS-“ or “ROS+” refers to spermatosoa without or with expression of ROS, respectively; ZEN: zearalenone; OTA: ochratoxin; T2: T-2 toxin; DAS: Diacetoxyscirpenol. The examined parameters included the following: Viable—the proportion of viable spermatozoa; ΔΨm—the ratio between the proportion of spermatozoa with polarized and depolarized mitochondrial membrane (log-transformed). Damaged and Intact acrosome—the proportion of spermatozoa with damaged or intact acrosome membrane integrity. Viable ROS- and ROS+—the proportion of viable spermatozoa without ROS or with ROS, respectively. Mixtures were made with the intermediate concentrations: 1 ppm ZEN, 0.25 ppm OTA, 0.1 ppm T2, and 0.1 ppm DAS, according to the binary mixture. The ‘Measured’ value is the actual outcome of each examined parameter obtained following exposure of spermatozoa to the ternary mixtures; the ‘Expected’ value was calculated for each examined parameter as the SUM of the effect obtained for three single mycotoxins. Presented are the values of the means ± S.E.M., which were adjusted relative to the control (i.e., with solvent). * *p* ≤ 0.05. Three interaction effects are suggested: (1) a synergistic interaction when the measured value was significantly higher than the expected value, (2) an antagonistic interaction when the measured value was significantly lower than the expected value, and (3) an additive interaction when the expected value was similar to that of the measured value (i.e., not significant).

**Table 6:** Concentrations of mycotoxins used in the study.

Mycotoxin	Concentration (ppm)	Concentration (µM)	Range of Admitted Limits in Animal Feed (ppm)
ZEN	0.1	0.34	0.1–3
	0.5	1.7	
	1	3.4	
	5	17	
	10	34	
OTA	0.05	0.124	0.05–0.25
	0.1	0.248	
	0.25	0.62	
	1	2.48	
	10	24.8	
T2	0.01	0.021	0.05–2
	0.05	0.107	
	0.1	0.214	
	0.5	1.07	
DAS	0.01	0.027	0.2
	0.05	0.136	
	0.1	0.272	
	0.5	1.364	

ZEN: zearalenone; OTA: ochratoxin; T2: T-2 toxin; DAS: Diacetoxyscirpenol.

## Data Availability

The data presented in this study are available in this article.
